# Interprofessional Education and the advances of Brazil

**DOI:** 10.1590/1518-8345.3148-3152

**Published:** 2019-04-29

**Authors:** Rodrigo Guimarães dos Santos Almeida, Cláudia Brandão Gonçalves Silva

**Affiliations:** 1 Universidade Federal de Mato Grosso do Sul, Instituto Integrado de Saúde, Campo Grande, MS, Brasil; 2 Ministério da Saúde, Secretaria de Gestão do Trabalho e da Educação na Saúde, Distrito Federal, DF, Brasil

The editorial The Interprofessional Education in Health in the Region of the Americas^(1)^, published in volume 26 of 2018 of this journal, addressed the commitment of the Pan American Health Organization (PAHO/WHO) to stimulate its Member States to support the formulation of policies that allow the expansion of Interprofessional Education (IPE)^(2)^ in the field of health teaching, thus strengthening Collaborative Practice (CP).

Given this scenario, it is important to highlight the initiatives already adopted by the Brazilian Ministry of Health through the Secretariat of Management of Labor and Health Education (SGTES in Portuguese).

In March 2018, the Secretariat of Management of Health Education (SGTES), in partnership with the Brazilian Network of Education and Interprofessional Work in Health (ReBETIS) and the Higher Education Institutions (University of São Paulo and Federal University of Rio Grande do Norte), together with PAHO, offered in the distance learning modality (DLM) the refresher course in Teaching Development for Interprofessional Health Education with the objective of qualifying approximately 300 professors regarding the theoretical-conceptual and methodological principles of IPE.

In July, the public notice of the Program for Education through Work in Health (PET-Health/Interprofessionality) was launched with the objective of promoting the teaching-service-community integration focused on the development of the Unified Health System (SUS), with a view to implement the IPE together with the Curricular Pedagogical Projects (CPP) of the undergraduate courses in the health area. According to the notice, up to 136 projects will be contemplated throughout Brazil with initiatives focused on Health Care Networks, with emphasis on strengthening Primary Health Care (PHC). The working groups will be composed of professors, students, and workers from the various health areas who, in a two-year period, will collaborate to implement IPE throughout the national territory, consolidating it as a macropolitical strategy.

The CP in health will only be possible if it is based on IPE strategies, in which students learn about, how, and with each other's work, from an understanding of common, specific, and collaborative practices that are articulated and enable, in an intentional way, the development of skills for collaborative teamwork^(3–4)^.

In the last decade, the adoption of more active methodological strategies and curricular changes have enabled changes in the training and health practices that are relevant to the dynamics of professionals' education. However, even in the face of scientific evidence of significant gains, there is still an incoherence between training and the needs required by the health system for teamwork^(5)^.

Thus, the adoption of policies that strengthen IPE may bring transformations to health practices, especially in the integration and collaboration among professionals, focusing on the health needs of users and the population, ensuring greater safety of care, reduction of errors by health professionals and costs in the health system. This may contribute to a strong SUS, capable of providing answers to the problems and health needs of the Brazilian population.

## References

[1] Silva FAM, Cassiani SHB, Freire JR (2018). Interprofessional Health Education in the Region of the Americas.
Rev. Latino-Am. Enfermagem. [Internet].

[2] Pan American Health Organization (2017). Interprofessional Education in Health Care: Improving Human Resource
Capacity to Achieve Universal Health.

[3] Peduzzi M, Norman IJ, Germani ACCG, Silva J AM, Souza GC (2013). Interprofessional education: training for healthcare
professionals for teamwork focusing on users. Rev Esc Enferm USP.

[4] Arruda LS, Moreira COF (2018). Interprofessional collaboration: a case study regarding the
professionals of the Care Center for Elderly, Rio de Janeiro State
University (NAI/UERJ), Brazil. Interface.

[5] Costa MV (2016). The interprofessional education in Brazilian context: some
reflections. Interface.

